# Supplementary material for: Energy deficiency selects crowded live epithelial cells for extrusion
Source: Nature. 2025 Sep 10;646(8087):1187–94. doi: 10.1038/s41586-025-09514-w (PMC12571905; doi:10.1038/s41586-025-09514-w)
Supplement: Supplementary file 1 — Table of inhibitors included in study. Inhibitor information is listed by commercial name, final concentration used for treatment of cells or PCLSs and the inhibitor target. [file 41586_2025_9514_MOESM1_ESM.pdf]

nature portfolio

https://doi.org/10.1038/s41586-025-09514-w

Supplementary information

Energy deficiency selects crowded live epithelial cells for extrusion

In the format provided by the authors and unedited

Inhibitor	Concentration	Target
4AP	1mM	Voltage-Gated Potassium Channels, e.g. $K_V1.1$, $K_V1.2$
Azimilide Dihydrochloride (Azi)	0.5µM	Voltage-Gated Potassium Channel $K_v11.1$ (hERG)
E4031	1 µM	Voltage-Gated Potassium Channel $K_v11.1$ (hERG)
Amiloride	10µM	Inhibitor of Epithelial Sodium Channel (ENaC)
Apamin	10µM	Small Conductance Potassium Channel (SK)
Tram 34	30µM	Intermediate Conductance Potassium Channel (IK)
Iberiotoxin	100nM	Big Conductance Potassium Channel (BK)
$GdCl_3$	10µM	Stretch Activated ion channels
GsMTx4	10µM	Piezo1, TRPC1, and TRPC6
CaCC inh-A01	10µM	Calcium activated Chloride channel
CFTR (inh)-172	5µM	Cystic Fibrosis Transmembrane Regulator (CFTR)
DCPIB	20µM	SWELL1/LRRC8A
Blebbistatin	100µM	Myosin II Inhibitor
Y27632	50µM	Rho kinase (ROCK)
H1152	5nM	Rho kinase (ROCK)
SKI-II	30µM	Sphingosine Kinase
SKI V2	4µM	Pan S1P receptor
JTE-013	10µM	$S1P_2$ receptor
Oligomycin A	20nM	Inhibits Mitochondrial ATP production
Oxamate	10µM	Glycolysis inhibitor
zVAD-fmk	100µM	Pan caspase inhibitor
