# Supplementary material for: Energy deficiency selects crowded live epithelial cells for extrusion
Source: Nature. 2025 Sep 10;646(8087):1187–94. doi: 10.1038/s41586-025-09514-w (PMC12571905; doi:10.1038/s41586-025-09514-w)
Supplement: Supplementary file 2 — Reporting Summary [file 41586_2025_9514_MOESM2_ESM.pdf]

nature portfolio

Corresponding author(s): Jody Rosenblatt

Last updated by author(s): Jul 31, 2025

Reporting Summary

Nature Portfolio wishes to improve the reproducibility of the work that we publish. This form provides structure for consistency and transparency in reporting. For further information on Nature Portfolio policies, see our Editorial Policies and the Editorial Policy Checklist.

Statistics

For all statistical analyses, confirm that the following items are present in the figure legend, table legend, main text, or Methods section.

n/a	Confirmed	
☐	☒	The exact sample size (*n*) for each experimental group/condition, given as a discrete number and unit of measurement
☐	☒	A statement on whether measurements were taken from distinct samples or whether the same sample was measured repeatedly
☐	☒	The statistical test(s) used AND whether they are one- or two-sided<br>*Only common tests should be described solely by name; describe more complex techniques in the Methods section.*
☐	☒	A description of all covariates tested
☐	☒	A description of any assumptions or corrections, such as tests of normality and adjustment for multiple comparisons
☐	☒	A full description of the statistical parameters including central tendency (e.g. means) or other basic estimates (e.g. regression coefficient) AND variation (e.g. standard deviation) or associated estimates of uncertainty (e.g. confidence intervals)
☐	☒	For null hypothesis testing, the test statistic (e.g. *F*, *t*, *r*) with confidence intervals, effect sizes, degrees of freedom and *P* value noted<br>*Give P values as exact values whenever suitable.*
☒	☐	For Bayesian analysis, information on the choice of priors and Markov chain Monte Carlo settings
☐	☒	For hierarchical and complex designs, identification of the appropriate level for tests and full reporting of outcomes
☐	☒	Estimates of effect sizes (e.g. Cohen's *d*, Pearson's *r*), indicating how they were calculated

Our web collection on statistics for biologists contains articles on many of the points above.

Software and code

Policy information about availability of computer code

Data collection	Commercial NIS Elements imaging Analysis Explorer package software version 5.41.02 Specifically the analysis explorer package was used to capture maximum IP (for z stack data to be analysed) by florescent channel then background estimates and mean intensity per entire image was captured. Further, the max intensity was used oh phase image to caputre HES and OICE raw data.<br>Excel (microscoft 16.89.1) Matlab version R2022a.
Data analysis	Fiji {lmageJ l.52p), Excel (microscoft 16.89.1), Commercial Graph Pad Prism software version 9.4.1, and Matlab version R2022a

For manuscripts utilizing custom algorithms or software that are central to the research but not yet described in published literature, software must be made available to editors and reviewers. We strongly encourage code deposition in a community repository (e.g. GitHub). See the Nature Portfolio guidelines for submitting code & software for further information.

Data

Policy information about availability of data

All manuscripts must include a data availability statement. This statement should provide the following information, where applicable:

- Accession codes, unique identifiers, or web links for publicly available datasets
- A description of any restrictions on data availability
- For clinical datasets or third party data, please ensure that the statement adheres to our policy

Due to their large size (total of roughly 24TB), raw microscopy data could not be made available but can be obtained from the corresponding author upon request. Source data are provided with this paper

Research involving human participants, their data, or biological material

Policy information about studies with human participants or human data. See also policy information about sex, gender (identity/presentation), and sexual orientation and race, ethnicity and racism.

Reporting on sex and gender	N/A
Reporting on race, ethnicity, or other socially relevant groupings	N/A
Population characteristics	N/A
Recruitment	N/A
Ethics oversight	N/A

Note that full information on the approval of the study protocol must also be provided in the manuscript.

Field-specific reporting

Please select the one below that is the best fit for your research. If you are not sure, read the appropriate sections before making your selection.

☒ Life sciences ☐ Behavioural & social sciences ☐ Ecological, evolutionary & environmental sciences

For a reference copy of the document with all sections, see nature.com/documents/nr-reporting-summary-flat.pdf

Life sciences study design

All studies must disclose on these points even when the disclosure is negative.

Sample size	The minimum sample size was determined according to the standards in the field and based on these calculations: Charan, Jaykaran, and N D Kantharia. "How to calculate sample size in animal studies?." Journal of pharmacology & pharmacotherapeutics vol. 4,4 (2013): 303-6.
Data exclusions	The only data exclusions were if MDCKII cellular monolayers were not dense enough to cause extrusion. Since extrusion is a mechanical regulated process of elimination areas or total cell counts were required to meet a density of ~60 cells per 100mm squared. Below this determined that not enough selective pressure lead to extrusion rates as previously described in published work.
Replication	Data was replicated minimum of 3 times by repeating the experiments on different days , in cell culture and different passage numbers. Further key experiments were repeated three times in an animal model using invivo mouse lung slices. All replications were successful.
Randomization	Extrusion, HES and OICE did occur in dense regions of the monolayers and since that was the mechanism being elucidated which were randomly choosen for analysis. The exception is when assessing the background cells and following extrusion. Cell in this case were identified for extrusion then followed back in time to determine the change in dyes and or phase microscopy changes.
Blinding	As much of the data analyses was done by the person running the experiments it was not possible to blind all analysis. However findings were confirmed with other author investigators blinded. Further most analyses and data collection depended on software capture of predetermined set parameters of florescence or white light from cell dyes and or genetically encoded probes or phase microscopy. Since the parameters were set based on controls the data collection was semi-automated and thus not collected with out bias.

Reporting for specific materials, systems and methods

We require information from authors about some types of materials, experimental systems and methods used in many studies. Here, indicate whether each material, system or method listed is relevant to your study. If you are not sure if a list item applies to your research, read the appropriate section before selecting a response.

Materials & experimental systems

n/a	Involved in the study
☐	☒ Antibodies
☐	☒ Eukaryotic cell lines
☒	☐ Palaeontology and archaeology
☐	☒ Animals and other organisms
☒	☐ Clinical data
☒	☐ Dual use research of concern
☒	☐ Plants

Methods

n/a	Involved in the study
☒	☐ ChIP-seq
☒	☐ Flow cytometry
☒	☐ MRI-based neuroimaging

Antibodies

Antibodies used

rabbit Piezo1 (Novus, NBP1-78446); mouse S1P (Santa Cruz, CA sc-48356); rabbit KCNA1 (Alomone Labs, APC-161); rabbit KCNA2 (Alomone Labs, APC-010); rabbit LRRC8A (Alomone labs, AAC-001); mouse ZO1 (Invitrogen, 33-9100); rabbit ENaC antibodies SCNNA1 (Invitrogen, PA1-920A), SCNNB1(Invitrogen, PA5-28909), and SCNNG1(Invitrogen PA5-77797). Alexa Fluor 488, 568 and 647 goat anti–mouse and anti–rabbit IgG were used as secondary antibodies (Invitrogen). F-actin was stained using either conjugated 488 or 568 phalloidin (66µM) at 1:500 and DNA with 1µg/ml DAPI (Thermofisher).Primary rabbit anti-E-Cadherin antibody (24E10, Cell Signaling 3195) at 1:1000.

Validation

When available the blocking peptides were utilized to provide a negative control. Further matching findings and temporal spatial information from previous published studies and commercial website images were used to set precedent of antibodies binding success. https://www.novusbio.com/products/piezo1-antibody_nbp1-78446;
https://www.scbt.com/p/edg-1-antibody-a-6?srsltid=AfmBOoof2AHM7OLfedK14dg0YC89JnzdivqHm1a-gJCOCk51NdO7UAEj;
https://www.alomone.com/p/anti-kv1-1-extracellular/APC-161; https://www.alomone.com/p/anti-kv1-2/APC-010;
https://www.alomone.com/p/anti-lrrc8a-extracellular-antibody/AAC-001;
https://www.thermofisher.com/antibody/product/ZO-1-Antibody-clone-ZO1-1A12-Monoclonal/33-9100;
https://www.thermofisher.com/antibody/product/alpha-ENaC-Antibody-Polyclonal/PA1-920A;
https://www.thermofisher.com/antibody/product/SCNN1G-Antibody-Polyclonal/PA5-77797
https://www.cellsignal.com/products/primary-antibodies/e-cadherin-24e10-rabbit-mab/3195?srsltid=AfmBOooDupf8T_CfuYrDGQQbU5w7wI9iV5cgBMF6ZpCR4kEQKij-v2--
Gudipaty, S. A. & Rosenblatt, J. Epithelial cell extrusion: Pathways and pathologies. Semin Cell Dev Biol 67, 132-140 (2017). https://doi.org:10.1016/j.semcdb.2016.05.010
Iorio, J. et al. K(V)11.1 Potassium Channel and the Na(+)/H(+) Antiporter NHE1 Modulate Adhesion-Dependent Intracellular pH in Colorectal Cancer Cells. Front Pharmacol 11, 848 (2020). https://doi.org:10.3389/fphar.2020.00848
Serra, S. A. et al. LRRC8A-containing chloride channel is crucial for cell volume recovery and survival under hypertonic conditions. Proc Natl Acad Sci U S A 118 (2021). https://doi.org:10.1073/pnas.2025013118
Eskandari, N. et al. Molecular Activation of the Kv11.1 Channel Reprograms EMT in Colon Cancer by Inhibiting TGFbeta Signaling via Activation of Calcineurin. Cancers (Basel) 13 (2021). https://doi.org:10.3390/cancers13236025
Sinha, M. et al. Chloride channels in the lung: Challenges and perspectives for viral infections, pulmonary arterial hypertension, and cystic fibrosis. Pharmacol Ther 237, 108249 (2022). https://doi.org:10.1016/j.pharmthera.2022.108249

Eukaryotic cell lines

Policy information about cell lines and Sex and Gender in Research

Cell line source(s)

MDCK-II: European Collection of Authenticated Cell Cultures (ECACC) operated by Public Health England, catalogue number 00062107, lot 19G037.

Authentication

MDCKII authenticated before shipping before shipping. Cells were not authenticated after reception.

Mycoplasma contamination

Mycoplasma contamination was tested monthly in the cell line. All cell lines and passages were negative for mycoplasma.

Commonly misidentified lines
(See ICLAC register)

no commonly misidentified cell lines were used in the study.

Animals and other research organisms

Policy information about studies involving animals; ARRIVE guidelines recommended for reporting animal research, and Sex and Gender in Research

Laboratory animals

Ex vivo lung slices were obtained from mice (B6N.129S6(Cg) - Scgb1a1 <tm1 (cre/ERT) Blh>J) from 7 to 17 weeks of age. Housed following guidance of King's College London project license P68983265 in ambient temperature (roughly 20C) keeping in normal a normal circadian rhythm.

Wild animals

No wild animals were used in this study.

Reporting on sex	No experimental design was implemented to test sex differences. Both male and female lungs slices were used.
Field-collected samples	No field collected animals were used for this study.
Ethics oversight	All animals were housed under specific pathogen-free conditions and cared for in accordance with the UK Home Office Animals (Scientific Procedures) Act of 1986 and the guidelines set by the Institutional Committees on Animal Welfare. Animal experiments received approval from the Ethical Review Process Committee at King's College London and were conducted under a Home Office license in the UK. All tissues collected for experiments were taken and preformed post-mortem. Following all approved and provided guidance from King's College London project license (P68983265) as per the Home Office.

Note that full information on the approval of the study protocol must also be provided in the manuscript.

Plants

Seed stocks	*Report on the source of all seed stocks or other plant material used. If applicable, state the seed stock centre and catalogue number. If plant specimens were collected from the field, describe the collection location, date and sampling procedures.*
Novel plant genotypes	*Describe the methods by which all novel plant genotypes were produced. This includes those generated by transgenic approaches, gene editing, chemical/radiation-based mutagenesis and hybridization. For transgenic lines, describe the transformation method, the number of independent lines analyzed and the generation upon which experiments were performed. For gene-edited lines, describe the editor used, the endogenous sequence targeted for editing, the targeting guide RNA sequence (if applicable) and how the editor was applied.*
Authentication	*Describe any authentication procedures for each seed stock used or novel genotype generated. Describe any experiments used to assess the effect of a mutation and, where applicable, how potential secondary effects (e.g. second site T-DNA insertions, mosiacism, off-target gene editing) were examined.*
